# Nurses’ experiences of ethical challenges concerning thirst in dying patients in specialist palliative care: a qualitative study

**DOI:** 10.1186/s12904-024-01519-y

**Published:** 2024-07-30

**Authors:** Maria Friedrichsen, Caroline Lythell, Micha Milovanovic, Nana Waldréus, Hans Thulesius, Tiny Jaarsma, Pier Jaarsma, Christel Hedman, Anne Söderlund Schaller

**Affiliations:** 1 Department of Health, Medicine and Caring Sciences, Linköping University, Linköping, Sweden; 2 Palliative Education and Research Centre, Vrinnevi hospital, Norrköping, Sweden; 3 Department of Internal Medicine, Vrinnevi hospital, Norrköping, Sweden; 4 Department of Neurobiology, Care Sciences and Society, Division of Nursing, Theme Inflammation and Aging, Karolinska Institutet, Karolinska University Hospital, Huddinge, Huddinge, Sweden; 5 Theme Inflammation and Aging, Karolinska University Hospital, Huddinge, Sweden; 6 Department of Medicine and Optometry, Faculty of Health and Life Sciences, Linnaeus University, Region Kronoberg, Kalmar, Växjö, Sweden; 7 Division of Society and Health, Department of Health, Medicine and Caring Sciences, Linköping University, Linköping, Sweden; 8 Department of Molecular Medicine and Surgery, Karolinska Institutet, Stockholm, Sweden; 9 R & D Department, Stockholms Sjukhem Foundation, Stockholm, Sweden; 10 Pain and Rehabilitation Centre, Department of Health, Medicine and Caring Sciences, Linköping University, Linköping, Sweden

**Keywords:** Ethical challenges, Nurses, Specialist palliative care, Thirst

## Abstract

**Aim:**

To describe nurses’ experiences of ethical challenges in relation to thirst in terminally ill patients in specialist palliative care units.

**Research design:**

A qualitative, reflexive thematic design with an inductive analysis was used.

**Participants and research context:**

Eighteen qualitative interviews with nurses working in six different specialist palliative care units in different hospitals in Sweden were conducted. The interviews were transcribed verbatim and analysed with a reflexive thematic analysis.

**Results:**

This study identified four themes that reflect ethical challenges experienced by nurses in the palliative care regarding thirst: Harmful infusions interfere with peaceful dying; conflict between tradition and personal experience; What is the right intervention to quench thirst? and; Lack of standard procedures, competence and interest among team members.

**Conclusion:**

Palliative care nurses experience a number ethical challenges in relation to thirst in dying patients. The main challenge is the provision of fluids to dying patients via artificial infusions, which nurses struggle with, as they do not want to interfere with a peaceful dying process.

## Background

Palliative care is the active, holistic and team based care of people of all ages with serious health-related suffering due to a serious illness, especially those nearing the end of life. It aims to improve the quality of life of patients, their families and carers [1]. A specialist palliative care provider may be a medical or nursing health professional who is recognised as a palliative care specialist by an accrediting body, or who works substantially in a specialist palliative care service where there is no accrediting body. The specialist palliative care provider have specialist knowledge, skills and expertise in the care of people living with a terminal illness and their families and carers, including the management of different complex symptoms, loss, grief and bereavement [2].

In palliative care nursing a humane approach to care delivery is required, as well as advocacy for the needs and comfort of patients [3]. Ethical sensitivity and perceptiveness are other vital skills in palliative care nursing practice [4]. Managing emotions of self and others, witnessing suffering and disability, caring for highly demanding patients and caregivers, as well as poor communication have been reported as distressing [5]. Therefore, it is important for nurses to have an ethical education and to continue to receive this education during their clinical work. Ethics training in nursing programmes varies around the world [6]. This is also the case in Sweden, where universities are relatively free to develop their own and local curricula for nursing programmes. However, the Swedish Higher Education Authority continuously scrutinizes them [7].

Nurses in palliative care need to provide comfort relating to a variety of health care needs of patients as they lose their functions, for example the function of drinking independently. As a consequence, thirst may occur in dying patients [8,9]. Thirst can be described as a deep sensation or desire for water that cannot be ignored and causes a powerful behavioural striving to drink water [10]. In the final week of a patient’s life, there is a risk of thirst as the patient may be unable to communicate his or her needs [11]. Previous studies on thirst were conducted 20–30 years ago, these studies viewed thirst as a cause of suffering for terminally ill patients [8, 9, 12]. Thirst studies are uncommon, as symptom scales used in palliative care usually measure dryness of mouth rather than thirst. An Australian study [13] compared regular oral care with mini mint ice cubes (1 cm^3^) containing 20% green mint syrup in a palliative care study population. The mini mints had a significant effect on both thirst and dry mouth. The difference between thirst and dryness of mouth may be difficult to distinguish. In one study, thirst was added to the Memorial Symptom Assessment Scale and it was significantly correlated with existential distress in terminally ill patients [14]. Nurses in palliative care are responsible for easing patients’ thirst, among many other symptoms. Thirst may therefore present an ethical challenge for nurses, especially when patients show signs of discomfort. However, there are no studies that present nurses’ views on the ethical challenges associated with thirst in specialist palliative care. This study is part of a larger project that studies how different professionals in palliative care experience thirst in dying patients and its related ethical dilemmas [15–17]. The project also studies the prevalence of thirst in palliative care patients (ongoing study) as well as the experiences of patients and family members [18]. The aim of the current study was to describe nurses’ experiences of ethical challenges in relation to thirst in terminally ill patients in specialist palliative care units.

## Methods

### Design

A qualitative, reflexive thematic design with an inductive analysis in accordance with Braun & Clarke [19] was used where coding and theme development are directed by the content of the data by shared meaning. The design aims to identify, analyse, and interpret patterns of meaning from the qualitative data, and to use the results to report concepts and assumptions underpinning the data, which are presented in themes.

### Sampling and setting

Data were collected during April 2021-January 2023 in six cities in Sweden, with populations between 3,000 and 1,000,000, all of which had specialist palliative care units. In Sweden, specialist palliative care is defined as care provided by multi-professional teams with specific knowledge and skills (education and experience) in palliative care [20]. These teams work with palliative care as their primary area of expertise, providing care directly to patients and their families and indirectly supporting other professionals in delivering such care. They address complex issues, which exist between different involved parties such as refractory symptom management and existential distress.

Specialist palliative care units provide home-based care and inpatient care at a hospital ward as well as consultation service. Nurses in palliative care handle a breadth of activities, in a holistic framework of understanding [21, 22]. In the Swedish health care system, all registered nurses (including nurses in palliative care) have a wide range of technical skills. These include giving injections, administering blood transfusions and other infusions, taking blood samples, inserting an intravenous line and enteral tubes and handling central venous catheters [23]. Swedish nurses provide respiratory suction, oxygen therapy, enteral nutrition and urinary catheterisation [24] They also use medical devices such as bladder scanners, pulse oximeters, patient-controlled analgesia pumps, nasogastric pumps, glucose and haemoglobin meters and electrocardiographs [23]. The role of the nurse in palliative care is also to assess and manage symptoms, communicate on different levels, and be a coordinator of care while using flexible and non-traditional methods in demanding and ethically challenged situations [21, 22].

A purposive sampling was used to achieve a diverse mix of participants based on geographic location, gender, age, and working experience in palliative care (Table 1). The inclusion criteria were fluency in Swedish and employment as a nurse in specialist palliative care. The head nurse of each department sent an invitation e-mail to eligible nurses, asking them to participate. The e-mail included a contact reference for a research nurse, if they were interested in participating.

**Table 1:** Demographic data of the participating nurses

**Gender**		
Female/Male	14/4	
***Age (years)***		
Mean Min-max	46 26–62	
***Work situation***		
Full time Part time	9 9	
***Experience working in palliative care (years)***		
Mean Min-max	13.1 1–38	
***Education***		
Bachelor of nursing Specialised in: *Anaesthesia* *Primary care* *Palliative care*	13 1 2 2	

### Data collection

Data were collected using individually recorded interviews (*n* = 18). The research team developed a semi-structured interview guide, used as a checklist to ensure that all questions had been discussed. One pilot interview was conducted to test the understanding of the questions and the flow of the questions. No changes were made after the pilot interview. The place, date, and time for the interviews were established in accordance with the nurses’ wishes. The majority of the interviews (*n* = 14) were carried out in privacy at the hospital in each city. Due to the COVID-19 pandemic, three interviews were conducted by telephone and one with Microsoft Skype©(Microsoft Corporation, California, U.S.A.). For the 18 interviews, two research nurses with an MSc, as well as experience with palliative care and interviewing, conducted two interviews each. A nurse lecturer with a Ph.D. in pain nursing management conducted six interviews. A medical student conducted six interviews and a nursing Ph.D. student conducted two interviews. All interviewers had a Master’s degree or Ph.D., which means that they had been trained in research methodology, which in Sweden includes interview techniques. The medical student had read research literature, but had only practised interviewing techniques on fellow students. All interviewers received practical instructions from the project manager before the interviews started. For those with a Master’s degree or undergraduate level, the interviews were reviewed with the project manager, who gave suggestions for further improvement as interviewers and possible follow-up questions. A professional transcriber transcribed all interviews verbatim. Each interviewer listened through their recordings so that they matched the transcribed interviews. The interviews addressed nurses’ experiences of caring for terminally ill patients and their views on thirst and related ethical issues [15]. They were reassured that the interview was not intended to “test” or check their knowledge. Rather, it was designed to seek their insights. They were asked to talk freely, and probing questions were occasionally asked in order to attain better clarity. In this study, ethical challenges were referred to as *ethical issues*,* moral challenges*,* moral dilemmas*,* values*,* good/bad*, and *right/wrong*. Ethical challenges could be labelled as such either by the authors or by the participants [25]. The interviews lasted between eight to 62 min. No new data emerged after 18 interviews, as the same codes and themes reappeared, but no new ones.

### Data analysis

A reflexive thematic analysis in accordance with Braun & Clarke [19] was used. The analysis started with familiarisation with the data, reading the whole dataset several times, generating initial codes of relevance to the research question, generating themes, reviewing, defining and naming themes. An example of the analysing process is shown in Table 2.

**Table 2:** Examples of the analysing process

Interview excerpts (interview number, page number and line number)	Generating initial codes of relevance to the research question	Generating subthemes	Defining and naming themes	Questioning and reflection process from co-authors	Themes of meaning Main theme and subtheme
*Then there can be a … special culture (to which the patient belongs) sometimes // I know on a few occasions that I’ve put in a subcutaneous drip // family members should know that the patient is getting something.17:9;8–9*	In some cultures, families want something to be done for the patient, in which case subcutaneous drips can be given.	Cultures values	Cultural values that require compassion drips	This quote may be more consistent with the text above (beginning of page 4). I don’t fully understand how the sub-theme follows the main theme, but it is a clear thread in the text. Nurses’ dilemma of balancing patient peaceful (death) with family needs and cultural values? I agree. I like the suggestion.	Main theme: Harmful infusions interfere peaceful dying Subtheme: Patients peaceful dying vs. family needs and cultural values
*It is the family members who…want us to give a drip// but in a way it is still the doctor who makes the final decision // It can be difficult at times*,* I think*,* to reach certain doctors // sometimes we get a bit on edge. // we have different opinions*,* different points of view. 13:9:19–23*	The family wants a drip. The nurse cannot always reach the doctors who make the decision as they have different opinions.	When nursing experience does not count	When nursing experience does not count	This quote is also informative in relation to the text. To be consistent with the main theme perhaps: When nursing experience does not count in End-of-Life infusions?	Main theme: Harmful infusions interfere peaceful dying Subtheme: Nursing experience does not count when deciding about infusions
*Someone who has like heart failure who has like a diuretic and who feels a great need that they want to drink but they are not allowed to drink. I:10;2*	Patients with heart failure are thirsty but not allowed to drink.	Ethical challenge where to draw the boundary in caring?	Where do you draw the boundary in caring?	This is open to multiple interpretations. Perhaps already, here state that they were not allowed to drink? Boundary challenges in navigating thirst? Finding ways to relieve thirst in the end of life?	Main theme: What is the right intervention to quench thirst?
*Then it tends to be*,* what should I say*,* that it is missed somewhat.// It has been a bit neglected before. I:8;3.*	The nurse misses thirst in patients; it has been neglected.	Thirst is neglected.	Neglect is a sign of disinterest.	Can this paragraph be included in one of the above texts describing the team, Clash between tradition and personal experience?	Main theme: Lack of standards, competence and interest among team members

Two authors, a PhD-student in palliative care nursing and a nurse with an MSc in palliative nursing care, separately made an analysis of the interviews. After this procedure, the first author (associate professor with a Ph.D. in palliative medicine and extensive experience in palliative care research) scrutinised the two analyses by comparing them with each other and with the interviews. The preliminary analysis was modified by, for example adding certain meaning, nuances, descriptions, and theme names to better explain the participants’ narratives. Data were analysed both manually, on paper (theme mapping), and in a Microsoft Word^®^ (Microsoft Corporation, California, U.S.A.) document (interview transcripts, coding). Additionally, the final analysis was examined and discussed in the research group until a consensus was reached.

### Ethical considerations

The study adhered to ethical principles outlined in the Declaration of Helsinki and was approved by The Swedish Ethical Review Authority (Ref. 2019–04347). Prior to the study, the nurses received oral and written information about the about the project, their ethical rights and who would be interviewing them.Written informed consent was obtained from all participants. There were no interdependencies between those interviewed and their interviewers. All collected materials in the study were handled confidentially and the participants were given a number to ensure confidentiality.

## Results

### Demographic data

Table 1 lists the demographic data of the 18 participating nurses. Of the participants, 14 were women and four men. The mean age was 46 years. Of the nurses, two were specialists in palliative nursing care.

**Fig. 1:**
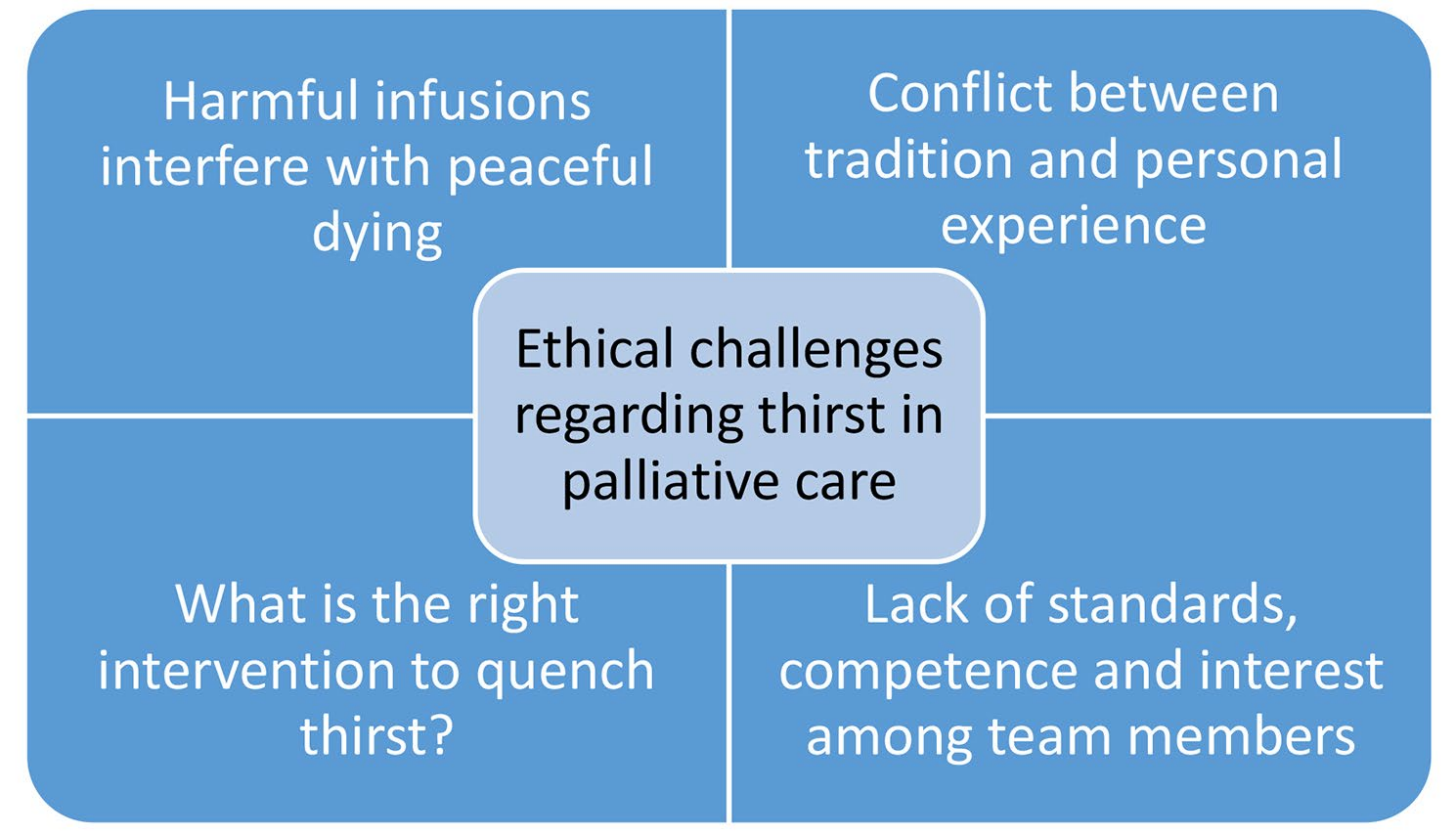
The four main themes in ethical challenges regarding thirst in palliative care

Four main themes of ethical challenges regarding thirst in palliative care emerged (Fig. 1).

### Harmful infusions interfere with peaceful dying

In this main theme, the following subthemes are included: Infusions becomes a burden to the patient; Patients peaceful dying vs. family needs and cultural values; and Nursing experience does not count when deciding about infusions. Nurses described how the most important thing to them was that they did not want to harm the vulnerable patient when he or she was dying; they wanted to give the patient peace during the dying process. Thirst was not a frequent topic of discussion in the teams’ day-to-day work. When it did come up, the focus tended to be on intravenous fluids at the end of life.

#### Infusions become a burden to the patient

Most nurses experienced that intravenous infusions did more harm than good to the patient at the end of life, and that they did not quench thirst. Nurses believed that the dying person’s body could only handle a limited amount of fluid. If too much fluid was administered, there was a risk of pulmonary oedema, which the nurses feared, as they did not want to harm the patient by causing breathing difficulties. Instead, their priority was to ensure that the patient could experience a peaceful last period with relieved symptoms. In addition, the procedure of inserting an intravenous line was considered a burden for the patient.

*This fear of causing a pulmonary oedema. That you are doing something wrong*,* that you are causing an increasing*,* more troublesome symptom than being thirsty. I:2*.

Some nurses had experienced that subcutaneous infusions were administered to patients at the end of life to quench thirst. They argued that even this infusion, which was initially considered less invasive, also caused harm to the patient by causing redness, swelling and pain locally at the injection site, which the nurses felt caused the patient distress. The nurses questioned the benefit of such an infusion.

*The family members were worried that he would die of thirst // then the doctors prescribed a (subcutaneous) drip // we thought it was an unpleasant pain that was inflicted on our patient*,* because you could see*,* often*,* the arm swelling or // yes*,* that it became red*,* it became irritated. I:17*.

#### Patients peaceful dying vs. family needs and cultural values

Nurses described that the most common reason why infusions were given to patients at the end of life was the wishes of family members. The encounters with persons from different cultures, i.e. from different countries, religions, occupations and health care cultures could cause ethical challenges for them, as they did not share the same goal regarding how end of life care should be carried out and how to quench thirst, as some family members from these cultures were more prone to request infusions.

Nurses described that most family members, regardless of which culture they came from, were worried about the patient drinking, as they wanted him or her to live a little longer, and had a fear that the patient would die of thirst. Sometimes this led to the prescription of, as the nurses put it, a “compassion drip”, i.e. an infusion that was given to make family members feel safe, even though nurses believed that the patient did not need it. Nurses claimed that they had to balance the best interests of the patient with the needs of the family, and they wanted to secure the patients a peaceful dying process.

*Sometimes we talked about a compassion drip because the family members feel that the patient is suffering from thirst // to reduce the suffering of the family members // … an ethical dilemma actually*,* in that we as healthcare professionals may see that a drip in this situation can worsen the patient’s condition. I:6*.

Instead, some nurses felt that an infusion often prolonged the dying process and the suffering of the patient while also sending contradictory signals to family members that there was a possibility of prolonging the patient’s life, when this was not the case. They had experienced how family members could find it difficult to accept when it was time to discontinue an infusion that the patient had been dependent on for a long time, as they saw a decision about the infusion as a choice between life and death.

*It is usually sensitive for a patient who has lived with a nutritional drip for a couple of months and has not been able to take in any other food and then you take it away; it can be associated with life and…and death. I:13*.

For the nurses it was obvious that the provision of infusion should not start when patients were at the end of life. However, they explained that continued hydration was of great importance to many family members in non-Western cultures to enable them to have faith and hope that a miracle could happen.

*It’s important in some cultures that you have it (IVs) so that you can be ready in case there is a miracle and then you have to have food and water*,* so there are things like that to take into account as well. I:2*.

Nurses described that in some non-Western cultures, the family members tried to feed or provide liquids to the dying patient in order to relieve thirst, even if the patient refused to drink. They did not respect the patient’s wishes but continued to give them fluids in order to save life, which made nurses feel frustrated as they felt that the patient could not die in peace.

*It can be a bit difficult with perhaps relatives and even staff sometimes*,* who try to think it is a good thing*,* and you may try to push fluids into the patient even though they do not want to them. I:9*.

Another culture could also be where family members of the dying patient work in acute health care, where this culture is used to treat and keep patients alive. This could also be demanding for the nurses, as they had to explain to the family members that the patient was dying and that different kinds of medical intervention would not help him/her anymore, but would instead cause harm.

*Not so long ago we had a man whose niece worked in emergency care. The man was no longer contactable*,* he was wheezing and had interruptions in breathing … The niece took it up with the patient’s doctor … and he prescribed an infusion … I:8*.

The nurses explained that they provided information to the family members that the infusion was no longer useful and would not quench their thirst; but was a burden to the patient. This information could help the family members to understand.

*I was thinking that some people want you to connect the drip…um*,* but…then we usually explain that giving a drip does not favour the patient’s sense of thirst…that it does more harm than good to… I:11*.

#### Nursing experience does not count when deciding about infusions

Nurses described how the team was divided in discussions about end-of-life infusions, with physicians being the ultimate decision makers. The nurses explained that physicians with less experience of palliative care found it difficult to refrain from infusions and listened more to family members than to the nurses, which made the nurses feel frustrated that their knowledge came second. They felt that their experience was not important, even though they wanted to protect the patient. Some nurses had also refused to provide an intravenous infusion in fear of causing more suffering to the patient.

*A colleague and I went in (to the patient) and judged that this… We do not do this; we do not carry out this prescription. Instead*,* we gave feedback to the doctor and described this patient’s situation. In addition*,* he*,* in turn*,* had to call the family members and say that this… this will not be good if we give this infusion. I:8*.

### Conflict between tradition and personal experience

The nurses described how their own experience and knowledge could be in conflict with the tradition of working with the symptom of thirst in palliative care. Nurses recounted that they relied on a traditional knowledge used in all palliative care, which emphasised that thirst did not occur at the end of life and that the body could not absorb fluids at the end of life. Instead, the fluids were deposited in other parts of the body such as the lungs and legs in the form of oedema. The nurses were accustomed to telling family members that patients were not thirsty, while at the same time doubting whether this was really the case. They themselves felt that the patient could suffer from thirst but that a strong practise in palliative care stated that the patient’s experience of thirst decreased at the end of life and that oral care was a sufficient measure to relieve thirst. This made it difficult for nurses to discuss the symptom of thirst within the team and they felt that the patient’s suffering might not be optimally alleviated.

*What we (in palliative care) say is that the patient is usually not bothered by thirst at the end of life. You don’t get hungry and you don’t get thirsty….we can certainly sense from time to time that patients are bothered by thirst. I:2*.

### What is the right intervention to quench thirst?

Several nurses felt that oral care was the only option to alleviate the experience of thirst in patients at the end of life. It was therefore a very important measure for symptom relief. An ethical challenge arose when it was necessary to consider the benefit versus the suffering of oral care, as the patient could sometimes experience it as very unpleasant, which the nurses felt was a difficult consideration. They did not want to take away the patient’s autonomy while wishing to carry out oral care to moisturise the mouth.

*Some patients who*,* even when they are not conscious*,* show*,* very clearly*,* strong discomfort from having someone who … yes*,* but moistens the oral cavity // then it becomes a small consideration // is it okay enough to skip this discomfort*,* or what is the greatest discomfort? It is very difficult if they cannot speak for themselves // What creates the most suffering? Not getting oral care or getting it done? I:14*.

There were also times when neither oral care nor infusions helped the patient who expressed strong thirst, which made the nurses feel unsatisfied, as these were the only tools they had. Then the nurses had to think outside the box and find new ways to quench the thirst, even if it meant taking time away from other patients.

*Yes*,* she (the patient) said she was thirsty*,* and we had a very hard time… because she rather threw up water … afterwards*,* we tried to sit there*,* just teaspoon by teaspoon. If you think a bit like after a stomach flu and so on when you just have to get the body to accept and retain some fluid. I:16*.

Some nurses differentiated between patients with different diagnoses, where patients with heart failure being the group they saw suffering most from thirst. The nurses described that it was a great suffering for these patients because they were thirsty but were not allowed to drink and quench their thirst due to fluid restrictions. This made the nurses feel frustrated, as the usual nursing advice in palliative care was not enough, such as sucking on ice cubes or drinking sips of water.

*Maybe then*,* I think it’s someone who has heart failure who has a diuretic and who feels a great need that they want to drink but they are not allowed to drink. I:10*.

Some nurses described that most patients at the end of life were heavily medicated and therefore assumed that they did not feel thirsty. However, it was difficult to determine whether it was right or wrong to give the patient such powerful drugs to mask other symptoms such as thirst.

*It’s a balancing act to find*,* should we just relieve*,* is there a cause that we can fix or should we just relieve in general and then I think with Morphine and Midazolam // that you calm // not sedate but kind of use those drugs also to remove symptoms like*,* like thirst. // that we*,* as I said*,* have a symptom that we only remove by giving Morphine and Midazolam…so are we doing the right thing? I:16*.

### Lack of standard procedures, competence and interest among team members

The nurses described that they felt that other health care professionals did not have knowledge or interest in whether the patients were thirsty. This led to the patient receiving poorer care, which the nurses found frustrating and an ethical challenge, as they did not want to see the patient suffer.

*That is the only problem in thirst that I see*,* an incredible amount of carelessness from the home care staff (municipality). We cannot give water often*,* the home care staff is there 6–8 times a day except for us who maybe are there max 2 times. Despite the fact that we write very clear plans*,* they do not often follow them and that is the biggest problem. I:1*.

Some nurses expressed that they themselves and other staff did not ask the patient if they were thirsty, which made them feel guilty and wonder if they had missed something. Nurses could also perceive oral care as a low priority, as they felt that many lacked interest in this even within their own organisation.

The lack of communication and forming common goals was perceived as ethically problematic. When the nurses described different ethical challenges around thirst, they described a situation where there is no standard procedure that they work in accordance with, which they found frustrating.

*Unfortunately*,* we are different people working*,* we have different goals in front of us*,* and we work differently. We have no*,* clear standardisation*,* which we all follow. This is a problem. I:1*.

## Discussion

This study identified four themes that reflect ethical challenges regarding thirst currently experienced by nurses in the palliative setting: Harmful infusions interfere with peaceful dying, conflict between tradition and personal experience, What is the right intervention to quench thirst? and, Lack of standards, competence and interest among team members.

In the theme “Harmful infusions interfere with peaceful dying”, nurses described how they wanted to give the dying patient a peaceful death, by not troubling the patient by giving infusions or inserting an intravenous line. This could be interpreted, as nurses being afraid of violating the patients’ dignity. Nordenfelt describes the dignity of identity [26], meaning that an intrusion in the private sphere is a violation of the patient’s integrity. Hurting a patient may involve a change in the person’s identity. Chochinov describes “The Platinum rule”, which would have health care professionals consider “doing unto patients as they would want done unto themselves”, for achieving optimal person-centred care [27]. In order to comply with the “Platinum rule”, all nurses must have a deeper and more mature relationship with the dying patient, which in today’s healthcare system may seem like a utopia. However, by using these theories, it becomes possible to understand nurses’ fear of disturbing a peaceful dying, as they seem to be aware what it means to interrupt in a dying process, i.e. violating the patient’s dignity as well as disturbing a peaceful dying process.

The subtheme “When nursing experience does not count”, describes when nurses feel that their experience was not utilised or requested, and that this was difficult for them to understand. In any case, the goal of palliative care must be to give everyone in the team a voice. The question of providing artificial hydration is a medical issue, even if nurses in Sweden are the professionals who are authorised to administer this task. The team must show unity towards the patient and their family, otherwise palliative care can be perceived as confusing, with different communication with the patient and with their family. This must be avoided as much as possible.

Within the theme “Conflict between tradition and personal experience” another common challenge for palliative care nurses was their experience of a loyalty conflict: the conflict between being loyal to the patient (not worsening his or her condition) and being loyal to the family members (reducing their suffering). Family members may request infusions to quench the dying patient’s thirst and in order to prolong the patient’s life. Nurses in palliative care believe that infusions become a physical burden to the dying patient. This experience of palliative care nurses is supported by other studies [28, 29], also palliative care physicians shared this belief [16, 30]. However, a review study [31] regarding artificial infusion reported the opposite, where six out of eight studies did not report higher respiratory secretions, and three studies out of four did not report dyspnoea. The scientific data conflicts with the clinical experience. There are many factors that may influence the clinical experience, such as statements by authoritative experts in a field, and old scientific studies that still influence the tradition of what should apply to palliative care. Two previous studies have shown that the tradition of withholding IV infusions in palliative care is strong [17, 32].

Another theme in the current study was “What interventions should be provided to relieve thirst”? Should nurses force patients to open their mouth so they could perform oral care or should they respect patient’s wishes? While respecting the patient’s wishes, preserving their autonomy, the patient would live his/her last days with an impaired oral health, which could be interpreted as a diminished dignity and quality of life. To respect the patient’s autonomy is important for nurses in palliative care worldwide [33]. According to the international code of ethics for nurses by the International Council of Nurses (ICN) [34], nurses are responsible for easing suffering and promoting a dignified death. Fundamental to nursing is also respect for human rights, including cultural rights, the right to life and freedom of choice, the right to dignity and to be treated with respect. Furthermore, the nurses needs to ensure that the individual and family receive understandable, accurate, sufficient and timely information on their own level [34]. However, this is difficult if the team’s opinions are divided on the issue. In this study, it was clear that nurses did not simply accept that physicians had decided whether a patient should receive a particular treatment, as there were nurses who described how they refused to perform a treatment that went against their own beliefs about what was right for the patient. This means that they rely on their self-esteem as nurses and their intuition.

However, ethical challenges are a matter of continuous education. At present, there are not set standards for what constitutes highly effective ethics education [35]. The content in such education should be related to common issues in clinical practice that cause moral distress and lead to ethics consultations.

## Conclusion

Palliative care nurses experience numerous ethical challenges in relation to thirst in dying patients. They are afraid of harming the patients’ autonomy as well as their dignity and they often experience a conflict of loyalty between supporting the patient and supporting the family, when the family member wants to quench patients thirst by giving them fluids or artificial infusions. It appears likely, that the nurses aim to achieve “the state of having fulfilled basic human needs”, thereby assisting patients in attaining dignity and promoting a calm and peaceful death.

### Clinical implications

**Fig. 2:**
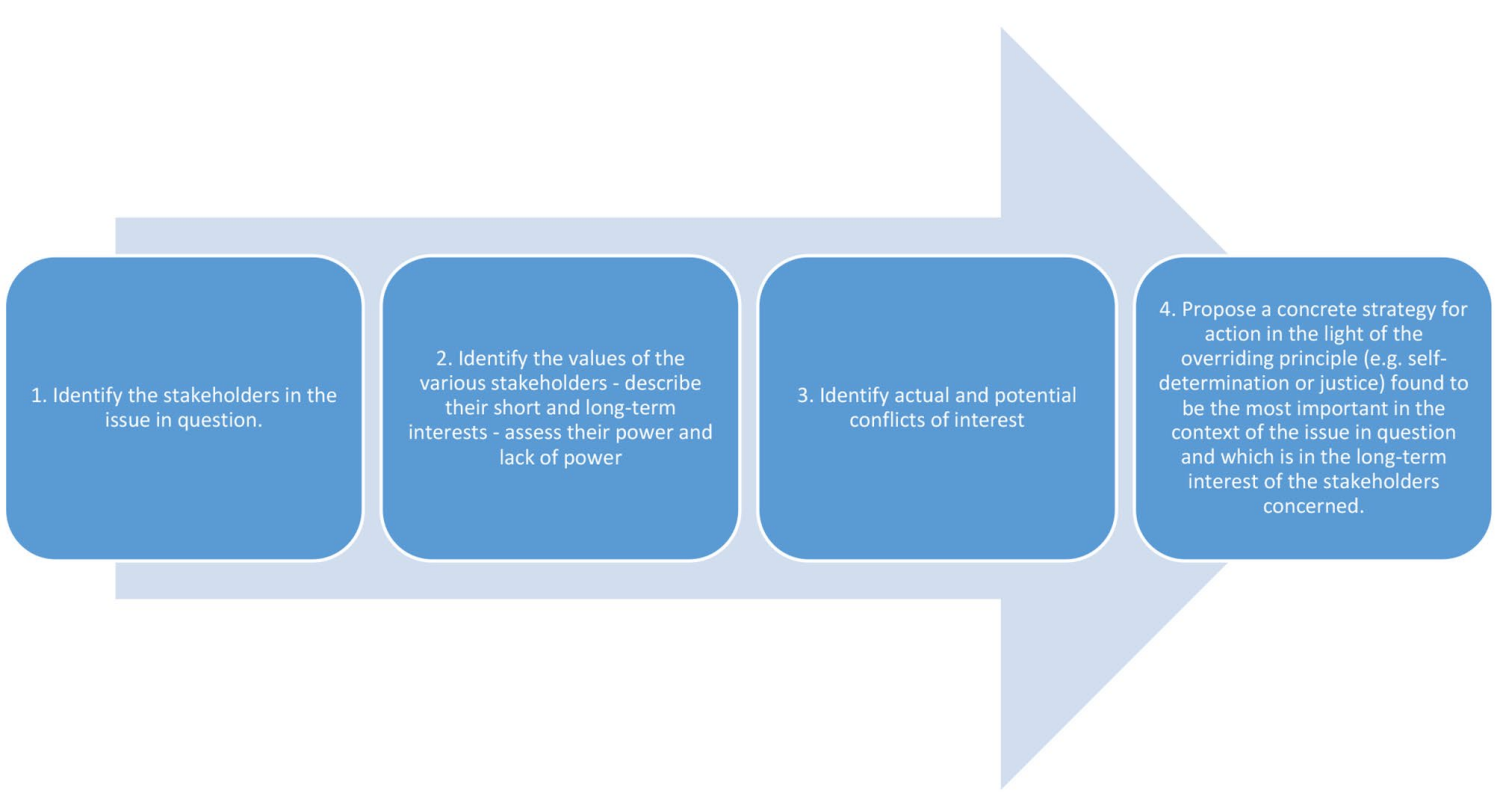
Ethical analysis developed by the Swedish National Council of Medical Ethics

Finding a solution to ethical challenges is an important issue in all practice. Even if the ICN Code of Ethics [34] is very clear and informative, it does not give clinical nurses help in every situation. An ethical analysis is needed to assess which ethical principle should be prioritised in each situation. Such an analysis was developed by Swedish National Council of Medical Ethics [36], and aims to identify the facts of the situation in question and to clarify underlying and sometimes invisible values, that can be helpful to nurses (Fig. 2).

### Strengths and limitations

One strength of this study was that we wanted to achieve a reliable coding and therefore used three different coders. We did this in order to test for consistency of judgement and also to sense-check ideas and to explore multiple assumptions or interpretations of the data aiming to achieve richer interpretations of meaning. Analytical transparency is accomplished by showing how the authors analyse and interpret data (Table 2). To enrich confirmability, we present quotations to support our study findings. We have tried to present the study context as precisely as possible for readers to decide on transferability of findings to their context. This study was conducted in Sweden and it is not obvious that it can be transferred and compared to other countries. A thematic analysis should not be equated with a content analysis. In reflexive thematic analysis, it is vital to conceptualise themes as patterns of shared meaning rather than as a domain overview of themes, which are only organised around a shared topic, as in content analysis.

## Data Availability

No datasets were generated or analysed during the current study.
